# Genetic, Environmental, and Stochastic Components of Lifespan Variability: The *Drosophila* Paradigm

**DOI:** 10.3390/ijms25084482

**Published:** 2024-04-19

**Authors:** Oleg V. Bylino, Anna A. Ogienko, Mikhail A. Batin, Pavel G. Georgiev, Evgeniya S. Omelina

**Affiliations:** 1 Department of Regulation of Genetic Processes, Laboratory of Molecular Organization of the Genome, Institute of Gene Biology RAS, 119334 Moscow, Russia; 2 Center for Precision Genome Editing and Genetic Technologies for Biomedicine, Institute of Gene Biology, Russian Academy of Sciences, 119334 Moscow, Russia; 3 Department of Regulation of Genetic Processes, Institute of Molecular and Cellular Biology SB RAS, 630090 Novosibirsk, Russia; 4 Open Longevity, 15260 Ventura Blvd., Sherman Oaks, Los Angeles, CA 91403, USA

**Keywords:** *Drosophila*, lifespan, longevity, ageing, genetic variability, phenotypic plasticity, stochastic variability

## Abstract

Lifespan is a complex quantitative trait involving genetic and non-genetic factors as well as the peculiarities of ontogenesis. As with all quantitative traits, lifespan shows considerable variation within populations and between individuals. *Drosophila*, a favourite object of geneticists, has greatly advanced our understanding of how different forms of variability affect lifespan. This review considers the role of heritable genetic variability, phenotypic plasticity and stochastic variability in controlling lifespan in *Drosophila melanogaster*. We discuss the major historical milestones in the development of the genetic approach to study lifespan, the breeding of long-lived lines, advances in lifespan QTL mapping, the environmental factors that have the greatest influence on lifespan in laboratory maintained flies, and the mechanisms, by which individual development affects longevity. The interplay between approaches to study ageing and lifespan limitation will also be discussed. Particular attention will be paid to the interaction of different types of variability in the control of lifespan.

## 1. Introduction

The fruit fly *Drosophila melanogaster* due to its short lifespan, has become one of the two principal model organisms for the study longevity and ageing, along with nematodes [1]. *Drosophila* has been used to study the genetics of longevity [2], cell signalling pathways associated with longevity [3], molecular mechanisms of ageing [4], the relationship between food quality, nutrition, and longevity [5], and much more.

**Figure 1:**
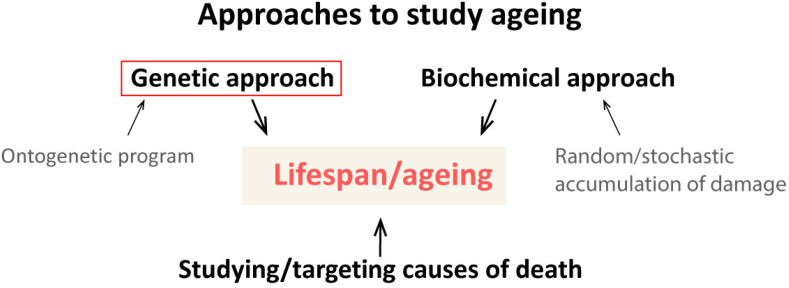
Three main approaches to the study of ageing. The genetic approach reflects the idea that ageing is an ontogenetic program and involves the searching for genes and gene variants (alleles) responsible for either increasing (longevity genes) or decreasing (ageing genes) lifespan. This approach also includes the study of the contribution of all types of variability (hereditary, lifelong phenotypic plasticity, and stochastic) to the control of the lifespan trait. The biochemical approach is consistent with the idea that ageing is a random accumulation of molecular damage with age (increase in entropy). This approach investigates the hallmarks of ageing, such as genomic instability, epigenetic changes, telomere attrition, loss of proteostasis, impaired macroautophagy, etc. An approach that studies the direct causes of mortality in organisms provides information about age-related diseases and health conditions that lead to the death of individuals. Medicine is concerned with targeting and preventing these causes of death in humans.

Ageing is a complex process affecting the lifespan trait, leading to impaired functions of cells, tissues, and organs and increased vulnerability of the organism to diseases and death. Ageing is on the list of unsolved problems in biology. Three main approaches to the study of lifespan and ageing can be discerned: genetic, biochemical, and the analysis of causes of death (Figure 1). The genetic approach generally aims to study how and why the value of any trait is varied or controlled. Therefore, it is crucial to understand what types of variability influence such a complex trait as lifespan. The quintessence of this approach is undoubtedly the identification of specific genes and/or gene variants that contribute to lifespan trait values. This approach correlates well with the idea that ageing, like development, is an ontogenetic program recorded in our genome [6,7,8,9,10,11,12,13]. The biochemical approach is devoted to studying the hallmarks of ageing, such as genomic instability, epigenetic alterations, telomere attrition, loss of proteostasis, disabled macroautophagy (mitochondrial dysfunction), etc. [14,15,16]. This approach fits well with the idea that ageing is a result of the random/stochastic accumulation of molecular damage [17,18,19,20]. Analysis of causes of death provides information about diseases and health conditions that cause the death of individual organisms [21,22,23,24,25]. This “medical” approach does not solve the problem of ageing but appears most promising from the perspective of delaying the death of individuals.

In this review, we describe in detail studies that have employed a genetic approach to investigate lifespan in the *Drosophila* model. We consider the role of genetic, stochastic, and environmental variability in controlling the lifespan of *Drosophila*. The history of the study of the lifespan trait in *Drosophila* from the beginning of the last century is also discussed.

## 2. History of *Drosophila* Ageing Research

### 2.1. First Steps in the Study of Drosophila Longevity

At the beginning of the last century, a number of interesting studies were conducted that opened up the history of ageing and lifespan studies in *Drosophila* and defined fundamental questions in the field of longevity research. For example, the important role of genetic background in controlling lifespan was first demonstrated in an experiment in which crosses between short-lived and long-lived lines of flies resulted in F1 hybrids that had a significantly longer lifespan and were superior to the long-lived parental line [26]. In addition, it was found that in some F2 hybrids of these lines, the lifespan tended toward the lifespan values of the short-lived line, symbolizing the effect of the return of the recessive genetic load [26]. The study also revealed sex differences in lifespan between males and females of the parental lines, and mixing F1 males and females in a population resulted in a significant equalisation of lifespan between the sexes, a finding that has since been replicated in numerous subsequent studies (see Section 3.5). Furthermore, the paper raised the question of the relationship between fecundity and lifespan for the first time. 

The groundwork for the study of the genetics of lifespan in *Drosophila* for years to come was laid in a series of studies by Prof. Raymond Pearl, who initially worked with flies bred by T. H. Morgan at the beginning of the last century. In particular, the following points were demonstrated:

- *Drosophila* quantitatively adheres to the same general law in the distribution of mortality as humans [27]. According to Prof. Pearl’s calculations, a comparison of lifespan between flies and humans revealed that 97 days in the life of a fly is equivalent to 86 years in a human (1 day in a fly = 0.8866 human years, or 1 year in a human = 1.1279 days in a fly) [28]. It was suggested that lifespan is a criterion that reflects the fitness of the organism [29].
- In populations of wild *Drosophila*, variability in lifespan exists, and if an inbred line is established from separate randomly selected males and females, the lifespan of flies from such a line is inherited across many generations [30]. These data have corroborated findings [26] that F1 hybrids between a short-lived and a long-lived line live longer than either parental line, and in the F2 groups of flies the characteristics of lifespan corresponding to the parental forms could be identified [29]. Thus, it was concluded that lifespan behaves like a typical Mendelian trait and, thus, it has a clear genetic basis.

It was also shown that a line combining five recessive mutations as homozygotes on one chromosome has an extremely low lifespan [29]. Furthermore, it was noted that there were no long-lived flies homozygous for the recessive *vestigial* mutation in any genetic background, and no group of flies with normal wings (wild type) exhibited the mortality curve characteristic of *vestigial* flies [29]. This was one of the earliest mentions of the detrimental effects of recessive homozygous mutations on lifespan. These results were confirmed using inbred lines with a homogeneous genetic background [31].

The results of Pearl et al. [32] were expanded upon in the very important work of Gonzalez et al. [33], who studied the effect of various recessive mutations as homozygotes and their combinations on lifespan. As a result of this work, it was established that individual mutations affecting the eye colour of flies or morphological characteristics of the body can influence lifespan. It was found that mutations can have not only negative but also positive effects on average, but not on maximum, lifespan (the effects of the *black* and *speck* mutations were studied individually). Furthermore, when combined, mutations that individually extend lifespan (*black* and *speck*) could, due to antagonistic epistasis, exert a detrimental effect on lifespan. Thus, for the first time, the potential for extending lifespan through genetic interventions and the interaction of mutations via epistasis was demonstrated.

The increase in average, but not maximum, lifespan demonstrated the effect of so-called “rectangularization” of survival curves. Despite the increase in average lifespan and the corresponding basic health of the population, the survival curve maintains its maximum values and takes on a rectangular shape [33]. This is apparently due to the inability to increase the maximum lifespan for a given genetic constitution and is, obviously, analogous to the limit to the maximum lifespan of the species. 

Additionally, in the study conducted by Gonzalez et al. [33], it was demonstrated for the first time that individual non-sex-linked mutations can affect lifespan differently in different sexes. The mutant lines exhibited varying numbers of progeny per female, sometimes surpassing those of the wild type. For instance, the *black* and *speck* mutants yielded fewer progeny than the wild type and displayed a longer lifespan compared to the wild type. Consequently, this raised the question for the first time about the influence of mutations on lifespan by altering the organism’s reproductive level.

In studies conducted during the same years, researchers also investigated the conditions for housing and feeding flies. We outline here the main findings of these studies, as they may be of considerable interest to researchers working with *Drosophila* and are crucial for experiments involving lifespan analysis using *Drosophila* as a model organism:

1. Flies can be anaesthetised with ether at least four times throughout their life without affecting lifespan [34].
2. Lifespan increases by 10% when bottles are covered with one layer of mesh bolting cloth instead of pieces of cotton wool [35].
3. The ability to fly increases lifespan—flies with removed wings live shorter lives than normal ones [32].
4. Under conditions of complete food deprivation, the lifespan of *Drosophila* is no more than 62 h in males and no more than 79 h in females. Under such conditions, there was no difference observed between the mutant (short-lived) *vestigial* flies and wild type (long-lived) flies, despite a three-fold difference in lifespan under normal conditions (when food is present). Variability in lifespan during fasting is reduced, including variability between males and females, with females being more variable in lifespan during fasting than males [36].
5. An increase in the number of flies from a few to 55 per one-ounce bottle causes an increase in the lifespan of the imagos, and as the number of flies in the bottle further increases, a decrease of up to 3.2 times in the imago lifespan is observed with a number of flies of 200 (linear regression) [37,38].
6. It is not possible to increase the lifespan of adult *Drosophila*/to rejuvenate adult flies by adding liquid extracts from chicken embryos or *Drosophila* larvae to the fly food [35].
7. The density of the reproductive population affects the number of offspring—the more flies lay eggs, the fewer offspring are produced per mated female [39].
8. The presence of an absorbent in the food (paper) does not affect lifespan [27].
9. The yeast is not essential for the nutrition of adult flies but is only required for the development of larvae and does not affect the adult lifespan. The lifespan of adult flies on agar with glucose, meat broth, and peptone (10 g/L peptone + 20 g/L glucose) is the same as on the same medium in the presence of 50 g/L of yeast [40]. Thus, peptone and meat broth can serve as substitutes for yeast in fly food.
10. On a synthetic minimal medium developed by Pearl et al. [41,42] containing only a set of necessary salts (MgSO_4_, CaCl_2_, (NH_4_)_2_SO_4_, KH_2_PO_4_) and cane sugar (83 g/L), the presence of sugar cannot compensate for the absence of yeast and the imago lifespan is significantly reduced compared to a medium containing yeast [43].
11. Extension of the larval period due to reduced nutrition does not lead to a lengthening of the adult (imago) lifespan but leads to a lengthening of the overall lifespan of the organism [43,44].

### 2.2. Investigation of the Role of Temperature in the Control of Drosophila Lifespan

In the same years, the effect of temperature on the *Drosophila* lifespan was studied in two similar classic works of Loeb and Northrop and Alpatov and Pearl [40,45]. 

The work of Loeb and Northrop [40] showed that the median lifespan of adults at 15, 25, and 30 °C is 92, 28, and 14 days, respectively. At 10 °C, adults live for 120 days, and the development of larvae is still possible, but pupae die at this temperature. The rate of larval and pupal development was found to be directly proportional to temperature, peaking at 28 °C, while the highest fertility, in terms of the greatest number of adults from laid eggs, was achieved at 25 °C. At temperatures above 28 °C, the development of larvae and pupae slowed down. Running longevity experiments at 28–29 °C can significantly increase adult mortality, and this technique is widely used in *Drosophila* longevity studies, allowing a complete survival curve to be obtained in one month instead of 2–2.5 months. In the same study, the presence of a temperature coefficient of mortality (where higher temperatures lead to faster fly mortality) was demonstrated. Additionally, the hypothesis was proposed that with increasing temperature, a substance is produced that contributes to ageing and death, or, conversely, under the influence of temperature, substances that normally prevent ageing and death are destroyed.

The work of Alpatov and Pearl [45] largely repeats the findings of Loeb and Northrop [40] and demonstrates that a decrease in the temperature where imagos are kept from 28 °C to 18 °C causes an increase in lifespan by 2.07 times, and with an increasing temperature, an exponential decrease in lifespan occurs. These observations established the rate-of-living theory, stating that a rise in temperature accelerates the rate of biological processes. At 18 °C, flies are very inactive and consume energy at a low rate during their growth and life, leading to lifespan extension. At 28 °C, flies are very active and have a shorter developmental period and rapid energy consumption, resulting in a shorter lifespan.

**Figure 2:**
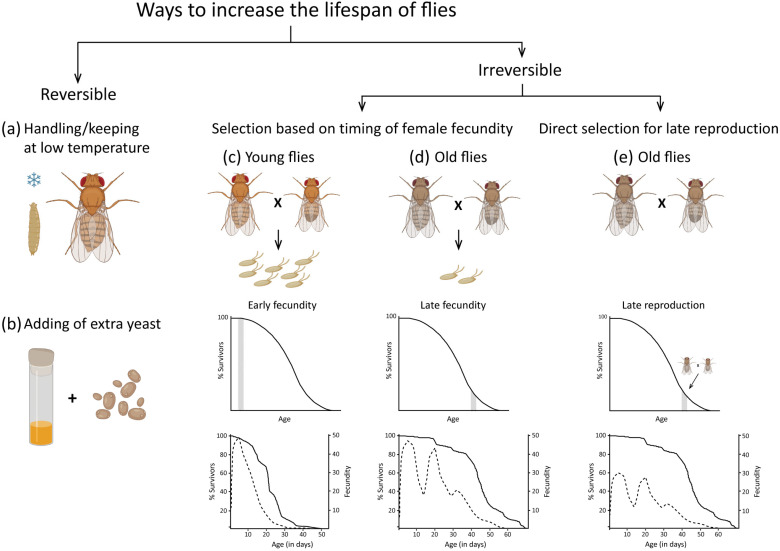
The ways to achieve reversible and irreversible increases in *Drosophila* lifespan. (**a**) Lowering the rearing temperature of larvae or adults is the simplest and most effective way to reversibly prolong the lifespan of *Drosophila* by reducing the rate of metabolic and biosynthetic processes (for details, see Section 2.2). (**b**) Yeast serves as a source of amino acids, carbohydrates, as well as B vitamins, niacin (also known as nicotinic acid, vitamin PP, or B3), folic acid, sodium, and potassium, and is essential for the development of larvae from non-wild type laboratory lines. Increasing their content in the fly food up to a certain threshold accelerates development and increases the imago lifespan, while exceeding the threshold reduces the imago lifespan (for details, see Section 2.3) (**c**,**d**) The selection of embryos from females at either early or late stages of their life (denoted by the vertical grey line) is accompanied by diametrically opposite effects on the lifespan of the imago (shown by the unbroken line, indicating the proportion of surviving flies) and the fecundity of females (represented by the dotted line). “Late” lines obtained after selection for late fecundity exhibit a second postponed peak of oviposition. (**e**) Direct selection for longevity is an alternative method to increase lifespan. In this approach, offspring are directly obtained from aged flies (both males and females). A potential side effect of this method is a decline in overall female fecundity, although this does not represent an absolute genetic correlation resulting from direct selection.

An extremely interesting observation was that adults whose larvae developed at 18 °C live significantly longer than adults whose larvae developed at 28 °C. These flies (18 °C) were larger in size and had more pronounced pigmentation on the dorsal side of the abdomen compared to the 28 °C flies [45]. When rearing imagos at 18, 25, and 28 °C, whose larvae developed at either 18 °C or 28 °C, it was found that at 18 °C and 25 °C, males (18 °C) lived significantly longer than males (28 °C) (well adapted), whereas at 28 °C, males (18 °C) lived slightly shorter than males (28 °C) (poorly adapted). The picture was somewhat different for females: at all rearing temperatures, females (18 °C) lived longer than females (28 °C) (well adapted). Thus, it was shown that not only did the keeping of imagos at low temperatures increase their lifespan, but also the development of flies at the larval stage at low temperatures prolongs the lifespan of imagos (Figure 2a). The effect of increasing body size was associated not only with an increase in cell size but also in their number [46].

The findings from the beginning of the century on the relationship between the rate of development and lifespan were further elaborated in later studies. For instance, it was confirmed that with increasing temperature in the range from 16 to 28 °C, the rate of larval development increases, and in the range from 28 to 31 °C, the rate reaches a peak and does not change [47]. However, the acceleration of larval development at elevated temperatures was accompanied by a decrease in lifespan (measured at 25 °C) and size of imagos, as well as female fecundity (the number of eggs laid), which was particularly affected by larval development at 31 °C [47] and was maximal when larvae developed at 25 °C [47,48]. Thus, it was determined that the lifespan of imagos decreases under the following conditions: (i) in response to accelerated metabolism with increasing temperatures from 16 to 31 °C and (ii) in response to accelerated larval development with increasing temperatures from 16 to 31 °C. Moreover, accelerated larval development due to temperature elevation reduces fecundity levels. 

The study of Cohet [49] showed that for the maximum longevity of the imago at 25 °C, there is an optimal temperature for larval development (17 °C), and larval development at temperatures within 12–14 °C results in a considerable reduction in the imago lifespan.

The study of Economos and Lints [50] re-examined the findings by Alpatov and Pearl [45] and Cohet [49] regarding the increased lifespan of imagos that developed at a lower temperature during the larval period (~18 °C). Larvae were maintained across a broader range of temperatures (19, 22, 25, and 28 °C), and lifespan was measured at all these temperatures. It was found that larvae that developed at 22 °C exhibited approximately the same lifespan at the imago stage at all studied temperatures (highly adapted). Larvae that developed at 19 °C, on the contrary, exhibited long adult lifespan only at 19 °C, while at 22 °C, 25 °C, and 28 °C, their lifespan sharply decreased (poorly adapted). Adult flies that developed at the larval stage at 28 °C displayed a shorter lifespan at all investigated temperatures, and those maintained during the larval period at 25 °C were intermediate between the data for 22 and 28 °C [50]. Thus, development at 19 °C reduces adaptability with a rise in temperature, development at 28 °C invariably guarantees a reduced lifespan across all temperature ranges, and flies developed from larvae maintained at 22 or 25 °C demonstrate greater resistance to upward or downward temperature changes. 

Differences among the experiments conducted by Alpatov and Pearl [45], Cohet [49], and Economos and Lints [50] may be attributed to variations in the experimental conditions employed by the different authors. For instance, Alpatov and Pearl’s study was conducted on an inbred line in 250 mL bottles, with 25 males and 25 females in each bottle. Cohet’s investigation, on the other hand, was carried out on F1 hybrids of *vestigial* and wild lines, focusing solely on females housed in plastic cages, providing the flies with the possibility of limited flight, with 15 females per cage. Meanwhile, Economos and Lints’ research utilized a mixture of four wild lines, focusing solely on males, housed in 250 mL bottles, with 50–100 flies per bottle. Moreover, all three studies employed different media to assess lifespan. Despite variations in experimental design and, to some extent, in results, the common theme across these studies is that decreased larval development temperature generally prolongs the imago lifespan. Moreover, larval development at an appropriate temperature may serve as an adaptation, enabling the flies to cope with temperature changes at the imago stage.

In summary, the studies on temperature have demonstrated the existence of non-heritable phenotypic variability (phenotypic plasticity) in adult lifespan, which can vary by approximately two-fold depending on the temperature at which the larvae develop (interaction with the environment).

The effect of shortened lifespan when flies were kept at elevated temperatures (27 and 29 °C) was found to be associated with the loss of ribosomes and mitochondria, as well as an accelerated accumulation of lipofuscin/ceroid (pigment bodies) in tissues. This phenomenon was observed particularly in post-mitotic cells, including those in the brain. Furthermore, there was more pronounced swelling and damage to neurons and glia in the brain compared to flies maintained at 21 °C [51,52].

Experiments on *Drosophila* at the beginning of the century, employing various temperature conditions, laid the groundwork for future research in the field of chaperones, autophagy, and proteostasis at large.

### 2.3. Relationship between Nutritional Composition and Lifespan of Drosophila

Experiments at the beginning of the century demonstrated that yeast is the main nutrient necessary for the development of *Drosophila* larvae. Yeast contains a large amount of protein, carbohydrates, as well as B vitamins, PP, folic acid, sodium, and potassium. In a series of studies in the 1980s varying the amount of yeast added to the food, the authors found a relationship between the duration of development, the larval growth rate (weight gain), and lifespan. Data from Alpatov and Northrop [43,44] confirmed this finding, showing that extending the larval period due to insufficient larval feeding does not lead to an increase in adult lifespan [53]. It was found that an increase in the body size of adults developed from larvae kept at a low temperature (18 °C) demonstrated by Alpatov and Pearl [45] is associated with greater amounts of yeast consumed by larvae at low temperatures compared to normal temperatures (25 °C) [54]. No increase in adult size was observed when the amount of yeast in the food was limited [54]. In contrast, at high larval densities, there was a decrease in adult body size at normal temperatures (25 °C) due to insufficient yeast in the food per larva [54]. With an increase in the yeast in the food, there was an increase in the size and weight of the adults, which, in turn, was accompanied by an increase in the number and size of cells, despite the fact that the rate of mitotic cell divisions remained constant [55]. The duration of development and the growth rate of larvae reached a plateau and ceased to increase beyond a certain concentration of yeast in the food [54]. Increasing the larval population density through the addition of extra egg portions while maintaining constant yeast concentrations reduced the development duration and the larval growth rate [54].

Adult lifespan was shown to be related to development duration, larval growth rate, body size, and cell number in a complex manner. All these values, including lifespan, increased to a certain threshold with an increasing concentration of yeast in the food (Figure 2b), but then, with a further increase in the yeast amount, they decreased [55,56]. This dependence of lifespan on body weight and the yeast amount was observed over a wide temperature range (19–29 °C) [50]. The lifespan was highest at the point when the growth rate of larvae, the number, and size of cells had not yet plateaued but were approaching that stage [55]. Thus, the increase in the rate of biological processes associated with cell growth and division, reaching a plateau at a certain point, begins to negatively affect lifespan. The maximum rate of development and growth due to increased feeding does not guarantee a long life. This may probably be due to an excess of incoming protein and other nutrients included in yeast (overeating negatively affects lifespan).

Later, the relationship between dietary protein/carbohydrate ratio and longevity, number of eggs laid during life, and egg production rate was studied in detail by Lee et al. [57]. The optimal performance for the three traits was achieved at protein/carbohydrate ratios of 1:16, 1:4, and 1:2, respectively. Thus, although excessive protein intake shortens the imago lifespan, it is necessary for egg production.

These studies conducted on *Drosophila* laid the groundwork for the extensive field of research on the impact of calorie restriction on lifespan using the *Drosophila* model. The first work on the connection between calorie restriction and lifespan in *Drosophila* was published in the early 1990s by Chippindale et al. [58]. It’s worth noting the remarkable fact that the era of studying the impact of calorie restriction on lifespan began in *Drosophila* much later than in mammals, where the first work in this direction was published in 1935 (see for discussion, for example, [59]).

### 2.4. Second Breakthrough: Selection for Lifespan in Drosophila

#### 2.4.1. Selection for Late Fecundity Increases the Lifespan of Flies

The next achievement in the genetics of longevity was the discovery of the possibility of controlling lifespan in an experiment, through the selection for longevity. The greatest contribution was made by the work of Michael Rose and his followers, performed in the 1980s. These works on selection were mainly performed on flies from wild populations with a high genetic diversity. The starting point for these studies was the demonstration of the fundamental possibility of selection for longevity, discovered for *D. subobscura* flies [60,61]. These works showed that natural populations of *Drosophila* have a reserve of allelic variants for the effective selection for an increased lifespan. 

In these studies, a methodology for such experiments was developed: selection of parents by age—young (on the 3rd to 10th day of life) or old (at 4–8 weeks of life). The eggs laid by older females were used to produce the next generation. Selection for longevity was performed for a maximum of 10 generations, and selection for early fecundity for a maximum of 30 generations. It was shown that the selection of eggs at early stages of life was accompanied by a decrease in lifespan and fecundity in flies, while the selection at later stages was accompanied by an increase in fecundity and lifespan (Figure 2c,d). These studies were followed up in the *D. melanogaster* model [62] and a negative correlation between early fecundity and lifespan, as well as between mean oviposition rate and lifespan, was found. Thus, early reproductive effort had a negative impact on lifespan.

Further to these studies, several classic works were performed by different groups in which selection for early and late fecundity was used. In a study of Rose and Charlesworth [63] over three generations, eggs were selected on days 19–20 of female life (lines “O”, Old) and on day 5 (lines “B”, Base, control). Selection for late fecundity reduced the early oviposition rate and increased the fly lifespan and the late fecundity of females subjected to selection [63]. These results were further developed in the work of Rose [64], where in the “O” lines eggs were selected over 15 generations starting from 28-day-old females at the beginning of selection and ending at 70 days of age at the end of selection, and in the work of Luckinbill et al. [65], where eggs were selected over 16 generations starting from 28 days of age at the beginning of selection and ending at 58 days of age at the end of selection. In the work of Rose [64], an increase in the lifespan of females was more significant than that of males and, paradoxically, was not accompanied by either a decreasing of early or late fecundity in the “O” lines, as might be expected. Thus, an increase in lifespan as a result of selection for late fecundity should not be accompanied by a decrease in overall or early fecundity. This is a very important result, showing that the classical disposable soma theory, which proposes a trade-off between reproduction and somatic stability [66], does not have to be followed. 

The opposite situation was found in the work of Luckinbill et al. [65]. In this paper, which also used a natural wild population of flies for experiments, the authors were able to see an almost two-fold increase in lifespan in response to selection for late fertility, as well as a clear connection between fertility and lifespan. Long-lived lines (“late” lines) had a second postponed peak of oviposition (Figure 2d), while control lines (“early” lines) obtained after selection for early fecundity had one early peak of oviposition (Figure 2c,d). Nevertheless, it is worth noting that in the work of Luckinbill et al. [65], flies after selection for early or late fecundity retained normal gradually decreasing fecundity until the end of life. Thus, although one might expect that when selected for late fecundity, the reproductive period of flies would last as long as that of flies selected for early fecundity, and subsequently flies selected for late fecundity would continue to live without producing offspring, this did not occur; the selection for both early and late fecundity did not make flies incapable of reproducing in the later periods of their lives. This clearly suggests that the longevity trait is strongly genetically correlated with the fertility/fecundity trait. Apparently, the better the overall health of an individual, the longer its reproductive period (although the total number of offspring may decrease—see below).

#### 2.4.2. Selection for Increased Lifespan Proceeds through a Phase of Inbreeding Depression

In addition to selection experiments on wild flies from natural populations, efforts were also made to select long-lived lines from a mixture of laboratory highly inbred wild type-like lines [67]. Eggs were collected on day 4 or day 26 of life for 13 generations. It was shown that selection for early or late fecundity is accompanied by a consistent decrease in lifespan approximately in the middle of the experiment (indicating an increase in inbreeding depression), followed by a subsequent restoration of lifespan to the original or slightly higher than the initial values (suggesting the selection of the most viable inbred variants). The maximum peak of fly oviposition selected for late fecundity was observed to be shifted 10 days later than that for flies selected for early fecundity [68]. The same authors later published a paper in which they failed to reproduce the data obtained from a heterogeneous wild type-like population on a laboratory highly inbred wild type line Oregon within eight generations of selection [69]. Thus, the initial genetic diversity of the population appears to be one of the conditions for successful selection for increased lifespan.

In the study of Luckinbill et al. [65], a similar observation was made: during both selection for early reproduction and selection for late reproduction, the overall fecundity of females decreased by the 17th generation of selection (fecundity was assessed within the range of 4–6 days after eclosion, as this period coincided with the time of selection for early fecundity), compared to flies that were not subjected to selection. Apparently, selection was also accompanied by inbreeding depression. F1 hybrids of “early” and “late” lines (both males and females) demonstrated averaged lifespan values, showing the recessive nature of the alleles responsible for increased lifespan [48,70]. The differences in early fecundity observed for the “early” and “late” lines were not found in F1 hybrids [48].

These data suggest that selection for late fecundity and increased longevity leads to the selection of generally more viable genotypes. The alleles responsible for increased longevity and postponed fecundity are recessive in nature, and the selection process goes through a phase of inbreeding depression, which could increase the frequency of such highly viable genotypes in the population, although the population still remains heterogeneous (see Section 2.4.6 for further details).

#### 2.4.3. Selection for Increased Lifespan Is Accompanied by the Changes in Morphology and Physiology of Flies

Further studies by Michael Rose showed that the “O” line flies had changes in morphology compared to the control “B” flies. Thus, the ovaries of females from the “O” lines were half the size compared with those of “B” flies [64]. Males and females of the “O” lines were more resistant to dehydration, starvation, 15% ethanol vapour, and heat shock at 37–39 °C than “B” line flies [71,72]. For both the “O” and “B” lines, resistance to starvation increased over the lifetime in females but was almost unchanged in males, whereas resistance to dehydration decreased in both sexes over the lifetime [71]. “O” lines were generally more resistant to starvation and dehydration than “B” at all ages [71]. The water content in “O” males was significantly higher than in “B” males [73] and conversely lower in “O” females than in “B” females [71]. 

Changes in morphology and stress tolerance in long-lived lines were associated with changes in physiology. Young females of the “O” line exhibited a lower respiratory rate and reduced locomotor activity compared to “B” females [74]. In aged flies, the difference in respiratory rate between “O” and “B” disappeared, and “O” females demonstrated greater locomotor activity. “O” flies showed an increased lipid content throughout their lives [72,74]. It is noteworthy that this content increased over the lifetime in both the “O” and “B” lines [74]. This may explain the increased resistance to starvation of “O” females with age, and the larger resistance of the “O” lines to dehydration and starvation in general. For “late” lines, changes in physiology were also observed [75]. The duration of continuous flight in flies of the “late” lines was 3–5 times longer compared with “early” lines; however, the dry weight of flies and body size did not differ between the “late” and “early” lines [75]. Increased flight duration was also found in the “O” line flies [72]. Moreover, “O” flies had increased tissue glycogen content [72], which is mobilized during both dehydration and flight. Likely, this contributed to the resistance of the “O” flies to dehydration and the prolongation of their continuous flight. Reverse selection of the “O” lines for early fecundity increased early fecundity and reduced the resistance to starvation of the “O” lines to the level of the control line; however, resistance to other stress factors, such as dehydration and ethanol vapours, did not change even after 22 generations of reverse selection [76]. Thus, the selection of long-lived phenotypes is accompanied by changes in morphology, physiology, and an increase in the stress resistance of flies. Apparently, some alleles in the “O” lines appear to be fixed, irreversibly increasing fly resistance to dehydration and ethanol, but frequency-dependent selection for other traits, such as fecundity or resistance to starvation, was still possible.

#### 2.4.4. Selection for Increased Lifespan Is Greatly Enhanced at High Larval Densities

An extremely important observation was that selection for long-lived genotypes was only possible under conditions of high larval densities, whereas selection for longevity was attenuated at low controlled larval densities [77]. Allowing females to lay eggs ad libitum resulted in a favourable response to selection. Conversely, if only a few larvae developed in the food, the response was weaker or absent. In addition, the density of larvae served as another factor that could interact with the genotype of the line. For instance, when “late” lines were reared at a low larval density, the lifespan of the adults dropped to the level of that of the “early” and “late” F1 hybrids reared at a high larval density [48,70]. In turn, the lifespan of the “early” and “late” F1 hybrids grown at low larval density decreased even further, reaching a level intermediate between the “early” lines and the “early” and “late” F1 hybrids grown at a high larval density [48,70]. Thus, larval density significantly contributes to the efficiency of longevity selection experiments. Even earlier, it was noted that the conditions of larval development could influence various physiological characteristics of imagos, including lifespan. For example, hybrids of two highly inbred lines reared at a high larval density exhibited increased lifespan and a prolonged reproductive period, including a postponed day of peak egg production, even in the absence of selection for longevity [78,79,80]. Nonetheless, weight, size, development rate, and mean egg production per day were diminished compared to adults developed at low larval densities [78,79,80], which was obviously due to inadequate nutrition at high larval density. Thus, even in the absence of selection for late fecundity, high larval density creates a situation where individuals with longer lifespans and prolonged reproduction periods predominantly develop. 

Interestingly, in studies by Lints and Hoste [67,68] and Lints et al. [69], low larval density was employed when selecting for longevity, and a response to selection from a genetically diverse population, while not very pronounced, was nonetheless achieved, whereas there was no such response when using a highly inbred wild type laboratory line at low larval density [69]. In the paper by Rose [64], selection from a genetically diverse population at low larval density resulted in a less robust response to selection compared to Luckinbill et al. [65], where both high genetic diversity and high larval density conditions were met. Moreover, under conditions of low larval density, reverse selection for early fecundity does not affect the “O” lines, which, at high larval density, results in an increase in early fecundity and a decrease in resistance to starvation [76]. Thus, selection for lifespan can be influenced by both the genetic diversity of the population and environmental factors, such as larval density in the culture and inter-larval competition. Each of these factors in the absence of the other can lead to an increase in the lifespan, but together they have a synergistic effect. 

The reason for the decreased effectiveness of selection for lifespan at low larval density is likely due to two main factors: (i) a decrease in phenotypic diversity (the narrowing of phenotypic plasticity due to reduced competition between individuals—decreased environmental pressure (the greater the environmental influence, the wider the reaction norm)) and canalisation (uniformity) of the phenotype and (ii) a reduced occurrence of beneficial stochastic variability due to the low number of individuals (see Stochastic variability section), involving stochastic variations in gene expression and stochastic advantageous ontogenetic variations, which can then be fixed in the genotype by new mutations (genetic assimilation process). Early studies found that wild, non-inbred lines of *Drosophila* show significant variation in both the rate of larval development across different stages and in their size (body length) [81]. On the other hand, the number of larval deaths increases as a function of population density, resulting in the survival of the most viable, largest larvae [82,83,84], which obviously possess better health and therefore the prerequisites for longevity (within the population, diversity in “individual robustness” exists, where the imposition of stressful conditions results in the survival of individuals that are healthier and live longer). Subsequent rounds of selection only consolidate this condition. At very high population densities of imagos and extreme larval densities, even in the absence of selection for longevity (adult flies in such conditions lived on average 2–3 weeks), the fecundity of females increased compared to females from a population with normal imago densities and relatively low larval densities selected for accelerated ageing and early fecundity (reproduction of these “early” flies was limited to a few days after emergence from pupae) [85]. Thus, a competitive environment appears to promote increased genetic health of the population and the removal of deleterious alleles, resulting in individuals becoming more fertile.

#### 2.4.5. Further Experiments, Biomarker of Death and Direct Selection for Increased Lifespan

With further research, several more attempts were made to select for long-lived *Drosophila* lines. The protocol described by Luckinbill et al. [65] was successfully replicated, demonstrating postponed fecundity and increased lifespan as reported by Arking [86]. The phenomenon of lifespan decline in the middle generations followed by a return to initial lifespan values in the final generations, as described by Lints and Hoste [67], was observed in both the control line where eggs were selected between the 2nd and 30th day of the females’ lives (no selection based on fecundity), as well as in the line with low larval density and selection for early fecundity. In all other cases (high density and early fecundity, low density and postponed fecundity, and high density and postponed fecundity), lifespan was increasing by the end of selection, and effects of high larval density or selection for postponed fecundity were observed. The most significant effect, similar to the original protocol, was achieved with a combination of high larval density and late fecundity.

This research also predicted a reliable biomarker for fly death: the elapsed time from the last fertile day (when larvae emerged from eggs) or from the last fecund day (when the last egg was laid) until death. This period of time averaged 10–12 days from the last fertile day and 7–9 days from the last fecund day (although there was a two-fold variability) and existed for both the long-lived line and the control line. An increase in the lifespan of a long-lived line seems to be due to the extension of the early and middle phases of the adult period of life, which results in the postponed onset of this biomarker that is characteristic of the later period of life. Thus, the physiological mechanism of mortality in flies may be similar to that which was recently found in *Caenorhabditis elegans* and may be associated with the end of the reproductive period [87,88,89,90].

Another successful attempt at developing long-lived lines was conducted by Partridge and Fowler [91]. They used two wild type fly strains from different parts of the world. Selection took place over 8–18 and 26–57 generations for the “young” and “old” lines, respectively. The eggs for the “young” lines were selected on days 7–14, while for the “old” lines, selection began on days 28–31 and extended to 70–73 days by the end of the selection. The resultant “young” and “old” lines showed no differences in fecundity and fertility early in life, but in the late period, “old” lines had significant advantages in both parameters, as well as increased lifespan. The developmental rate from egg to adult and the larval development rate under different density conditions were lower for “old” flies compared with “young” flies. The morphological characteristics of the “old” flies (thorax length) did not differ from those of the “young” flies. However, the wet weight of the “old” flies was greater than that of the “young” line flies. Thus, selection in the “old” lines led to a longer lifespan, reduced early fecundity, extended reproductive period, and morphological changes, all of which had already been reported previously. What was novel was that all these changes were accompanied by an extension of the period of growth and development, which had not been observed before. Partridge and Fowler [91] did not control larval density, which was high for both “young” and “old” lines, and the selection results, though notable, were less dramatic than those in work of Luckinbill et al. [65] more closely resembling the outcomes reported by Rose et al. [64]. The “old” and “young” lines were further studied by Roper et al. [92] and similar data were obtained as in the other experiments described above. For instance, in the “old” lines, inbreeding depression was detected, which disappeared in the F1 hybrids. Neither the F1 hybrids nor the original “Base” line exhibited an increase in lifespan or late fecundity, coupled with a reduction in early fecundity. This suggests the recessive nature of the alleles responsible for these effects.

Another method for breeding long-lived lines has been the direct selection approach [93,94,95,96], utilising various wild type lines from nature [94], highly inbred laboratory lines [95,96], and a mix of several non-inbred laboratory lines [93]. In this strategy, eggs from long-lived females were not selected (in this situation, only females actually live long, and the male may be much younger, having mated with a female some time ago and died), and offspring were obtained directly from crosses between males and females that lived to the required late age. This approach has been successful for both selected males [93,94,95,96], and females [94,95,96], over long and short selection periods [94]. Interestingly, long-lived female lines generally produced fewer offspring [94] (Figure 2e) or a comparable number to control lines [96], but no increase in fecundity during later stages of life was observed in long-lived lines [94,96]. 

Varied outcomes concerning changes in physiology and stress tolerance were observed following direct selection by different research groups. For instance, long-lived lines showed an increased resistance to starvation and a higher lipid content [94], which is a trend observed in other experiments employing selection for late fecundity [72,74]. In the experiment of Deepashree et al. [96], more parameters of long-lived flies were assessed. Resistance to starvation and dehydration was unexpectedly reduced in females but remained unchanged in males [96]. This contradicts the findings from studies by Service et al. [71,72] regarding the starvation resistance of long-lived flies after selection for late fecundity. At the same time, direct selection increased cold tolerance in both sexes and enhanced locomotor activity throughout life, but this effect was observed only in males [96]. Regardless of the noted effects on resistances to starvation and cold, lipid content in long-lived flies under direct selection did not differ from control flies [96], which is also inconsistent with the data [71,72]. The discrepancies in outcomes may be explained by differences in the mode of selection or the distinct genetic backgrounds of the populations used for selection by different research groups. The identification of long-lived lines with enhanced antioxidant protection in flies represented a new finding compared to previous studies [96]. Despite the declining activity of superoxide dismutase (SOD) and catalase over the lifespan, and the increasing levels of reactive oxygen species (ROS) observed in both long-lived and control flies, long-lived flies, both males and females, exhibited elevated SOD activity throughout their lives. However, catalase activity and glutathione (GSH) content were significantly increased only in long-lived males [96].

Thus, the results from direct selection for longevity are in line with experiments on selection for late fecundity. Long-lived lines maintain increased stress resistance (at least against certain stresses and in at least one sex), and an elevated lipid level does not appear to be an essential requirement for stress resistance in long-lived flies. Crucially, the direct selection approach enabled separation of the selection effects on late fecundity from those on increased lifespan.

#### 2.4.6. Lines Selected for Increased Longevity Maintain It for Decades and Have a Non-Inbreeding Genetic Population Structure

Re-examination of the “O” lines for the presence of characteristic features, performed 10 years after the initial breeding, showed the preservation of all features at the same level [97]. Flies that are selected for longevity have a longer lifespan than flies that are caught in the wild [98]. This is significant because it challenges the notion that selection for longevity in laboratory settings merely restores the lifespan potential inherent in wild, genetically diverse flies, which had been compromised in laboratory strains due to inbreeding depression. Importantly, both the “O” and “B” lines, after selection, were found to be heterogeneous rather than completely inbred, indicating the potential for further selection based on specific traits of interest [99]. The selection for longevity continues to the present day, and the “O” and “B” lines are utilised in biotech start-ups (https://longevity.technology/news/do-long-lived-flies-hold-the-key-to-extending-human-longevity/amp/, accessed on 17 April 2024). 

Furthermore, interest in researching these lines persists. The genomes of the “O” and “B” lines were sequenced, revealing 6394 genetic differences across 1928 genes between these lines [100]. The 2.6 Mb at the end of the X chromosome contained the largest number of fixed alternative polymorphisms, indicating a positive selection for mutations in this region of the genome. Males and females exhibited differential expression in 175 and 98 genes, respectively, that appear to be associated with postponed ageing and fecundity [100]. The transcriptome was also determined in these lines [101], and experiments were conducted to study the influence of genes active in germinal tissue on lifespan [102]. Further studies, including the production of transgenic *Drosophila*, will be required to elucidate the molecular details of how individual gene groups affect lifespan in these lines.

#### 2.4.7. Selection for Lifespan May Be Accompanied by a Variety of Genetic Correlations

**Figure 3:**
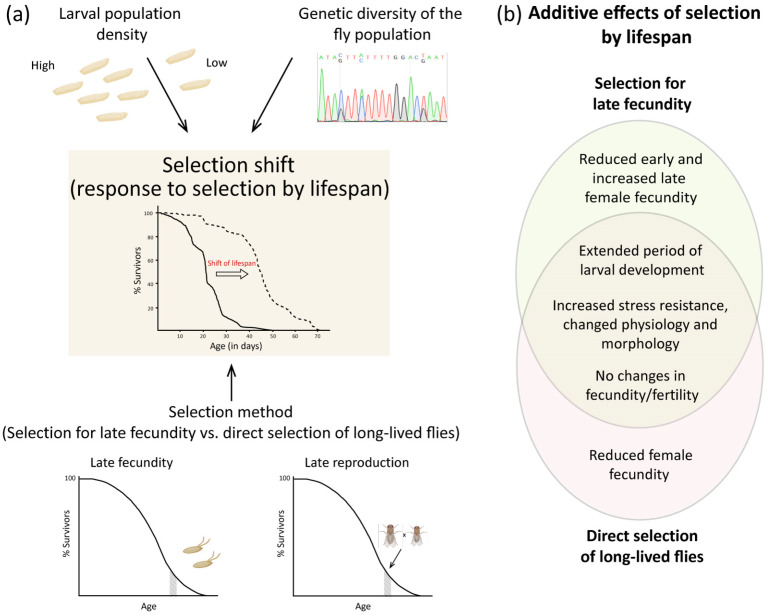
The primary factors influencing the effectiveness of selection for lifespan and the main genetic correlations accompanying the selection. (**a**) The primary factors influencing the effectiveness of selection for lifespan include: the density of the larval population, the initial genetic diversity of the fly population used for selection, and the method of selection (choosing between selection for late fecundity and direct selection for longevity). The most significant effect is achieved by combining the density of the larval population and the initial genetic diversity. The selection method determines the presence of a delayed second peak of fecundity (in the case of selection for late fecundity). Direct selection for longevity does not result in such a second peak (or produces a less pronounced second peak) (see Figure 2). (**b**) Possible genetic correlations that have emerged during selection for increased lifespan are presented. None of the genetic correlations are absolute, except for some stress resistance.

Thus, numerous attempts have been made to successfully select flies for increased longevity. In various experiments, the response to selection (the selection shift) for the lifespan trait differed. It depended not only on the selection method (selection for postponed fecundity, direct selection of long-lived flies), and its duration but also on additional factors, such as larval population density (an environmental factor) and the initial genetic diversity of the fly ancestors’ population (Figure 3a). As the experiments utilised natural populations of wild flies from diverse origins, the qualitative and quantitative composition of genetic diversity (i.e., the frequency of certain alleles, as well as a set of mutations fixed in the population) could significantly influence the extent of the selection shift in different experiments. Increases in lifespan as a result of selection for late fecundity could either be accompanied by a decline in early fecundity and an increase in late fecundity of females, or it might not be accompanied by any fecundity changes (Figure 3b). Moreover, selection for late fecundity, as well as direct selection for longevity, could lead to an extended period of larval development. Under direct selection of long-lived flies, an increase in lifespan could be accompanied by a decrease in female fecundity or occur without any change in fecundity levels. Lifespan and reproduction level appear to influence each other yet remain independent traits. At the same time, selection for longevity was often accompanied by an increase in stress resistance (at least to some stressors), changes in the physiology and very often even the morphology of the flies (Figure 3b). Thus, selection for lifespan may exhibit genetic correlations with other traits; however, lifespan often can be dissociated from those traits, except for stress resistance, which tends to be consistently present to some extent.

#### 2.4.8. Initial Stages of Speciation in Lines of Long-Lived Flies 

The breeding of a long-lived line, accompanied by an increase in the lifespan of individuals and a change in their reproduction pattern, morphology, and physiology can be considered as the initial stages of speciation. A change in allele frequencies, rather than the fixation of specific mutations, is characteristic for evolutionarily young, recently formed species and accompanies the stages of early speciation [103]. Hence, it is not surprising that after selection for longevity, lines are characterised by genetic heterogeneity, and further selection for traits of longevity and fecundity is possible [99] (https://longevity.technology/news/do-long-lived-flies-hold-the-key-to-extending-human-longevity/amp//, accessed on 17 April 2024). Further selection for longevity in fly lines should eventually lead to partial reproductive isolation of “late” lines from “early” or “Base” lines.

#### 2.4.9. Heritability of the Lifespan Trait in Flies

To understand how important the heritable component of lifespan is in *Drosophila*, a comparison with humans is appropriate. In a heterogeneous human population, the heritability of the lifespan trait is ~10% [104]. The heritability of longevity in monozygotic twins is ~18–35% [105,106,107,108,109], with the heritability in monozygotic twins being twice as high as in dizygotic twins [109,110] and decreasing dramatically under different twin living conditions, demonstrating the strong influence of environmental factors on longevity [109]. Meanwhile, the heritability of longevity (people over 100 years old, centenarians) can be as high as 48% for men and 33% for women [111]. According to Perls et al., siblings of long-lived individuals are 17 and 8 times more likely, respectively, to reach the same age than the population average [112]. This means that human longevity may be associated with genetic variants that are rare in the population [113,114,115], although variants that are common in the population are also important for longevity [116,117].

In *Drosophila*, the heritability of lifespan is 6.5–9% within a line [107,118]. An attempt to clarify these data using the classical technique of generating F1 hybrids between long-lived and short-lived lines, as well as F2 hybrids, to determine the heritability of lifespan has yielded ambiguous results [119]. Deepashree et al. concluded that the heritability of the increased lifespan trait in the long-lived lines depends on genetic background, environmental factors and maternal effects [119]. The authors attributed the lack of heterosis in F1 hybrids to low genetic diversity in the parental inbred lines, although in the early 20th century the experiments of Pearl et al. showed that in F2 it was possible to distinguish groups of flies with lifespan characteristics corresponding to the parental forms [32]. Thus, the heritability of lifespan appears to be lower in *Drosophila* than in humans. It seems that non-genetic factors and stochastic variability play a significant role in the control of lifespan in *Drosophila*.

To summarise, it can be emphasised that the simplest way to achieve a reversible increase in the lifespan of *Drosophila* imagos is by maintaining the larvae or adults at low temperatures (Figure 2a). Another factor that reversibly increases the lifespan of imagos is the elevated level of yeast in their diet (Figure 2b). An irreversible increase in lifespan is achievable through the selection of eggs from older females (selection for late fecundity) (Figure 2c,d), or by breeding offspring from older, long-lived parents (direct selection for longevity) (Figure 2e). The outcome of selection can be markedly enhanced by a high larval density (by augmenting the range of phenotypic plasticity, or by selecting the individuals most resistant to stress), as well as due to the high genetic diversity within the population (Figure 3a).

## 3. The Role of Hereditary Variability and Environmental Factors in the Control of *Drosophila* Longevity

Phenotypic traits of an organism, from simple ones such as gene expression to complex and quantitative ones such as lifespan, develop during ontogeny as a result of the interaction of genetic and environmental factors [120,121]. Phenotype variations, the phenotypic variability of individuals, depend on the following factors:

(i) Heritable or genetic variation expressed through the heritability of a trait (including lost heritability—rare variants that have a significant impact on the value of the trait) [122,123];
(ii) Non-hereditary or non-genetic (phenotypic) variability (phenotypic plasticity or reaction norm (dispersion) of a trait, the ability of the same genotype to produce different phenotypes depending on environmental conditions) arising in response to changing environmental conditions throughout life [121];
(iii) Stochastic variability in ontogeny (peculiarities of ontogeny and random fluctuations in individual development, commonly referred to as ontogenetic noise) [124] and the associated stochastic variability in gene expression.

**Figure 4:**
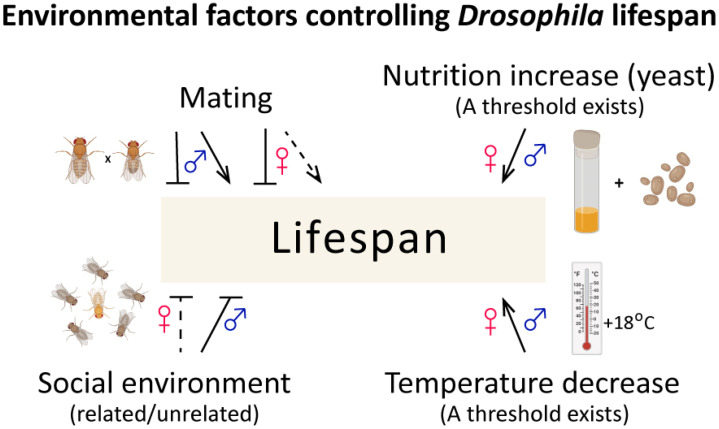
The influence of environmental factors on the lifespan of laboratory cultures of *Drosophila*. Positive effects are indicated by an arrow, while negative effects are indicated by a blunt arrow. The influence of temperature and diet is discussed in Section 2.2 and Section 2.3.

When keeping *Drosophila* in laboratory conditions with a fixed temperature, humidity, and light cycle on a rich nutrient medium, the influence of environmental factors on the lifespan is minimised. In such cases, the role of hereditary and random variability comes to the fore. The influence of the stochastic component of variability on lifespan is the subject of a separate discussion, and there are quite a few experimental results on this issue obtained with *Drosophila*. In this part, we will first consider at what stage the study of the genetic component of lifespan control in *Drosophila* is. Then, we will discuss the influence of some environmental factors, such as mating and social environment (the influence of nutrition and temperature is discussed in the history section, see also [125]), on lifespan (Figure 4), and finally, we will briefly touch on the stochastic component of variability.

### 3.1. Genetic Polymorphism in Drosophila Populations

Identification of genes and specific variants of genes (alleles) that control lifespan and ageing is essential for understanding the differences in lifespan between individuals in natural populations, as well as the reasons for lifespan limitation in different species (“species lifespan barrier”). Genetic polymorphism in natural populations is a powerful reflection of the action of evolutionary factors or forces, including mutation, gene flow, genetic drift, and natural selection on the trait of lifespan. Generally, natural populations of *Drosophila* are characterised by a high level of DNA polymorphism [126,127,128], including loci that affect lifespan. On average, NGS sequencing data indicates that the nucleotide substitution rate in individual lines is approximately 1 per 305 nt [129]. In studies of protein polymorphism conducted between the 1960s and the 1980s, it was found that *Drosophila* populations from different regions generally exhibited similar levels of polymorphism [130]. Within individual populations, up to 69% of the examined loci were polymorphic, with an average level of polymorphism between populations of 30–40%. An individual fly in a population is heterozygous, on average, by 10–15%, with a maximum of up to 23% [130,131,132,133,134,135,136,137]. These findings are generally in agreement with more recent results obtained through sequencing, which suggest that 77% of the examined protein-coding genes in inbred lines have at least one amino acid substitution [138]. However, according to two-dimensional electrophoresis data, polymorphism for the most highly represented proteins in *Drosophila* was extremely low [139], which indicates the effect of strong purifying selection on *Drosophila* ‘housekeeping’ genes.

Positive selection for mutations that increase fitness and their fixation in the population was accompanied by a decrease in genetic diversity and a decrease in the level of genetic polymorphism in the vicinity of the fixed mutation [140]. Importantly, the level of protein polymorphism in large populations (5000 individuals) maintained in laboratories for many years corresponded to the level of polymorphism in natural populations [132]; however, in small populations (500 individuals or fewer), genetic diversity rapidly decreased (a pronounced effect is visible over 50 generations) [141]. In the experiment, when switching from a standard feeding environment to other dietary conditions, genotype frequencies for genes associated with food assimilation quickly changed for all studied loci (population of 2500 individuals, 16 generations) [142]. This demonstrates the high adaptability of *Drosophila* to new environmental conditions and underscores once again the possibilities of rapid selection in this model organism.

Natural populations of *Drosophila* were also remarkably resistant to environmental and pharmacological influences. For instance, none of the 19 environmental, pharmacological or physical influences, except for lowering the temperature to 18 °C, was able to significantly increase the lifespan of flies [143]. Moreover, surprisingly, an increase in temperature to 28 °C, which usually greatly reduces the lifespan of laboratory lines, had a much less pronounced effect on the lifespan of wild flies from natural populations, indicating their high resistance [143]. 

Thus, natural populations of *Drosophila*, with their substantial genetic diversity, represent an inexhaustible source of genetic variation and material for selection, including for studies on lifespan and longevity.

### 3.2. Genetic Polymorphism Affects Gene Expression

How does natural genetic polymorphism contribute to differences in lifespan between individuals? Natural genetic polymorphism in *Drosophila* populations leads to variations in gene expression [144,145] at the level of individual tissues [146,147,148], during development [149,150,151], and in response to stress. Many polymorphisms are expression quantitative trait loci (eQTLs) [152,153]. The overall transcriptome variability in a panel of 192 inbred lines was estimated to be 42% [128]. It is evident that this set of polymorphic sites in these lines does not exhaust all possible genetic diversity in natural populations of *Drosophila* in the wild. Moreover, considering the combinations of epistatic interactions between polymorphisms affecting gene expression, transcriptomic variability may be even broader.

Since inbreeding in laboratory lines of *Drosophila* is achieved through sibling mating, and starts, as a rule, with one fertilised female caught in the wild, and taking into account the average level of heterozygosity of individuals in nature of 10–15%, the variability in lifespan in laboratory lines of *Drosophila* appears to depend on a relatively small number of genes. In line with this, a large portion of gene expression variability may be insignificant or only weakly significant, and variability in only a small fraction of genes may determine substantial differences in the lifespan of individuals. Indeed, the transcriptional response to selection for longevity in long-lived “O” lines with postponed fecundity and delayed ageing, achieved by selection for late fecundity, involved changes in transcription of only 6% of the investigated loci (age of the studied flies—10% and 90% of mortality), compared to control lines “B”, where no selection was performed [101]. 

The transcriptional response to selection for longevity in long-lived “O” lines has been extensively studied by Wilson et al. At an early age (the age of the flies studied was 10% of mortality), the response to selection included activation of genes for proteases, proteins involved in redox reactions, genes related to mitochondria, electron transport chain, endoplasmic reticulum, microsomes and vesicles, and genes involved in detoxification of xenobiotics, indicating the importance of rapid active metabolism and stress resistance at a young age. At the same time, genes responsible for immune responses to bacteria and fungi, muscle function (contractile fibres, sarcomeres and myofibrils), cytoskeleton (actin) genes and genes involved in polysaccharide catabolism, aminoglycans and sugar biosynthesis were activated in the ‘B’ lines, indicating the detrimental effects of increased inflammation, muscle function and sugar degradation/synthesis at an early age on longevity [101].

In old “O” line flies (age was 90% of mortality), the genes for proteases, phosphatases, folic acid metabolism and xenobiotic detoxification were activated compared to the “B” line flies, whereas the genes for components of lipid particles, contractile fibres, sarcomeres and myofibrils were activated in the “B” flies. In addition, in old “B” flies, the expression of some proteases, phosphatases, β-galactosidase, methylene-TGF dehydrogenase and some neurogenesis genes changed with age, whereas in “O” flies the expression of these genes did not change from young to old (corresponding to the young age of “B” flies) and remained stable (signature of delayed ageing). Moreover, some genes that were down-regulated in old age in “B” flies were up-regulated in “O” flies compared to young age in “B” flies (proteases and phosphatases) and, conversely, genes involved in immunity, catabolism/anabolism of aminoglycans and polysaccharides that were activated in old age in “B” flies were down-regulated in “O” flies. 

Thus, active metabolism and stress tolerance in late life appeared to ensure a longer lifespan and reproductive period, whereas excessive muscle function, reduced protein metabolism, inflammation, lipid accumulation and activation of sugar degradation/synthesis in late life were associated with short lifespan [101]. A ‘younger’ pattern of gene expression in lines selected for longevity has also been noted by other authors [154]. A notable finding of Wilson et al. is that among the genes found to be altered in expression in “O” flies compared to “B” flies, and thus associated with increased longevity and lengthening of the reproductive period, there are no genes or candidate genes previously identified as genes associated with *Drosophila* longevity. These results highlight the importance of detecting and characterising genetic variation in nature as an alternative approach to de novo mutation analysis.

The transcriptional response of the genome to ageing (age of flies studied—90% mortality) consisted of changes in the expression of 19% of genes, i.e., it was broader than the response to selection for longevity [101]. These figures are roughly in line with previous data on the transcriptional response to ageing (17% of all genes) [155], (23%) [156], (33%) [157], but lower than estimates by other authors (7%) [158]. The trajectories of transcript representation change markedly during the life of flies, with expression of some genes changing very strongly and others not significantly [156]. The transcriptional signature of ageing included decreased activity of genes related to proteolysis, metabolism, nucleotide synthesis, oxidative phosphorylation and mitochondrial function, indicating a general decline in metabolism with age. In contrast, genes related to protein synthesis, defence against bacteria and fungi, xenobiotic detoxification, cell cycle, cytoskeleton, aminoglycan and sugar catabolism, and amine biosynthesis were activated in old flies, indicating a potential compensatory mechanism for the age-related decline in metabolic activity [101]. Other authors have observed a decrease in the transcription of genes in germinal tissue, genes related to muscle function and the cytoskeleton, as well as genes related to proteolysis, metabolism and transport of substances into the cell [158], and, in contrast, increased expression of immune genes [156,158], pro-apoptotic genes [159], heat shock proteins and antioxidant defence proteins [158]. Xenobiotic detoxification genes showed a variable pattern, i.e., some decreased in expression, others increased, but in general more P450/Cyp genes increased in expression with age than decreased [158,159].

Thus, the transcriptional signature of ageing reflects a general decline in metabolism, motor activity and reproductive functions with age, but with apparently activated mechanisms that attempt to compensate for the general decline, but for some reason this is accompanied by over-activation of the immune response and pro-apoptotic genes, increasing the fragility of the organism with age.

### 3.3. Lifespan QTLs

#### 3.3.1. The Current State of Research in the Attempt to Identify QTLs Associated with Lifespan

Not so many studies have been conducted to search for QTLs affecting longevity in *Drosophila* [160]. Initial genetic analysis of lines subjected to selection for late fecundity and increased lifespan (“late” lines) showed that lifespan can be controlled by a single factor [161]. Further analysis of the chromosomal localisation of such factors revealed that longevity is under polygenic control, and the genetic determinants responsible for increasing lifespan in “late” lines are located on the 1st and 3rd chromosomes, with the greatest contribution of loci located on the 3rd chromosome [162]. 

When studying the chromosomal localisation of lifespan QTLs of a long-lived line of another origin (NDC-L), it was also found that lifespan is mainly determined by recessive QTLs on the 3rd chromosome [163]. Although, in addition to these QTLs, there were several more QTLs on other chromosomes that interacted with each other epistatically. In contrast, using crosses between long-lived “O” and short-lived “B” lines, it was found that longevity QTLs were partially dominant, and a minimum of 10 lifespan QTLs were identified on the two major autosomes [164]. When mapping using microsatellite analysis, three major lifespan QTLs were found in the “O” lines, although many markers responded to selection of different directions [165]. Thus, the common theme of the studies was the idea that the number of longevity QTLs is relatively small; however, the genetic history of the lines and the methodology used in the research influenced the number of QTLs detected.

Subsequent studies aimed at identifying longevity QTLs conducted in several genetic models using recombination mapping [166,167,168] and deletion mapping [169,170,171] generally confirmed the initial ideas that not many loci are involved in the control of lifespan in *Drosophila* (up to 19 according to various estimates). However, large QTLs were shown to be broken down into smaller QTLs, and each individual QTL covers a large number of genes [172,173,174]. To localize the genes responsible for lifespan, these QTLs were subjected to complementation tests with mutations, and as a result, the first genes affecting lifespan were found [170,175,176,177]. 

Later, using genome-wide association studies (GWAS) and large panels of sequenced inbred lines derived from wild fly populations, no definitive results were obtained [178], and using the same panel of fly lines, Huang et al. [2] showed that about 1000 genes were associated with variations in lifespan in *Drosophila*, although other estimates using the same panel of lines linked only 52 SNPs to lifespan [179]. Using another similar panel of lines, it was found that longevity in *Drosophila* was associated with only 8 loci [180], or, according to other authors, five QTLs, each of which encompassed 11–155 protein-coding genes [160]. Thus, although polymorphism of individual ageing-associated genes do contribute to variations in the lifespan in wild fly populations [170,175,181,182,183], there is no clear understanding of how many longevity QTLs exist.

Moreover, it has been discovered that in some nature populations, along with QTLs associated with increased lifespan, detrimental QTLs increasing mortality at an early age or at all ages were also detected at a low frequency [184]. Additionally, “silent” QTLs potentially capable of affecting lifespan under some other conditions, for example in combination with other (“permissive”) mutations, were found [185]. Interestingly, many of the detected QTLs also showed pleiotropic effects, i.e., they affected not only the lifespan of flies but also female fecundity/fertility, stress tolerance, and also reduced the basic mortality in lines [168,186,187,188,189]. Thus, it is conceivable that longevity QTLs in natural fly populations influence lifespan through mechanisms similar to those of genetic determinants derived from selection experiments, with both impacting the basic health of fly lines.

#### 3.3.2. The Problems and Constraints Associated with QTL Analysis

The QTL approach has been extensively criticized by some researchers due to the possible high number of false positives and epistasis potentially influencing the results [190]. Many QTL markers may in fact correspond to mobile genetic elements scattered throughout the genome [191].

An important issue in the effectiveness of QTL analysis is the validation of the genes discovered. Typically, candidate genes predicted at QTL loci are validated in the same investigations using RNAi knockdown [2,102], and only a few of these candidate genes have been subsequently validated in separate studies [175,177,181,192,193]. Furthermore, even when a set of polymorphic nucleotides is known for validated candidate genes, determining the mechanism by which a specific nucleotide affects lifespan is very challenging [181]. In such cases, researchers typically confine their analysis to the association of these nucleotides with the phenotype [181,192]. Detected gene variants are never transferred to another genetic background, and there are only a few examples where the effects of polymorphisms on gene expression identified in natural populations have been experimentally tested using genetic constructs [192,194]. Additionally, it is not uncommon for a specific SNP to be tightly linked to a trait, despite demonstrating a weak effect on the expression of the candidate gene, even in the presence of a strong correlation between the gene’s expression and the trait [195]. This may be an “enhancer” SNP, in addition to which, due to strong linkage disequilibrium, there is another “causal/key” SNP that plays a major role [195]. In these instances, the mutual influence of various mutations on the deleteriousness or usefulness of each other occurs, i.e., epistatic interactions between alleles [196,197]. Also, the case of simple additive genetic variation is also possible; that is, a simple, combined, independent effect of polymorphisms/changes in the expression of several genes on the lifespan trait [163,198]. All these problems significantly reduce the significance of the results of QTL identification and essentially arise from the chosen genetic approach.

The model of genetic control of quantitative traits suggests that there is a large number of loci, whose polymorphisms/mutations make a small contribution to the value of quantitative traits, including lifespan [120,199,200,201]. Numerous epistatic interactions occur between such individual polymorphisms (antagonistic, synergistic, and other forms of epistasis) [197] (possible forms of interaction between two factors are illustrated at https://synergyage.info/methods//, accessed on 17 April 2024). However, precise mapping, even of individual polymorphisms, let alone deciphering the molecular mechanisms of their influence on a trait, can take up to 20 years [202,203], and when it comes to identifying combinations of polymorphisms, such investigations can span several decades, often exceeding the active working period of an individual researcher. 

Modern sequencing technologies do not provide clarity, as authors admit that even with huge panels of sequenced inbred lines derived from wild populations, there is insufficient statistical power to accurately identify the individual genes responsible for changes in lifespan, and even more so the “guilty” genetic variations within these genes [178]. 

Furthermore, despite numerous assertions regarding the role of epistasis in lifespan control, no direct experiment demonstrating the mutual influence of two polymorphisms on lifespan has been conducted, possibly because such an experiment would require the transfer of two variants of non-allelic genes into a specific genetic background, although CRISPR technologies would allow such an experiment to be carried out. Despite the apparent unambiguity of the results of such an experiment and numerous examples of mutual associative influence between different DNA polymorphisms, its outcome is far from obvious, as the effect of a specific genetic background on a particular pair of interacting polymorphisms cannot be predicted.

Thus, it is necessary to acknowledge that we are still far from identifying a specific set of DNA variations responsible for increasing lifespan, even in such genetically tractable organisms as *Drosophila*. The number of such variations and their qualitative compositions remain unclear. Initial claims of a relatively small number of variations responsible for changes in lifespan have, with further research, evolved into estimates exceeding 1000 variations [2]. The immense effort expended on searching for the association of DNA polymorphisms with lifespan, and the modest validated results ultimately obtained, indicate the need for alternative, new approaches to identifying specific genomic DNA sequence changes that control lifespan. The development and validation of such approaches might well be a priority for the forthcoming decades.

### 3.4. Sexual Dimorphism and Lifespan Control

Sexual dimorphism in *D. melanogaster* arises from essentially the same genotype (X0 males develop normally but are sterile), as a result of intricate mechanisms regulating gene expression. It is evident that sexual differences, including sets of genes differentially expressed in males and females, will influence lifespan. Sex differences in lifespan are characteristic of all well-studied species, and *Drosophila* is no exception in this regard [204]. Polymorphism in both the X chromosome and autosomes affects the lifespan of both *Drosophila* males and females [198].

Some studies have shown that males exhibit greater variability in lifespan than females, despite potentially greater genetic variability in females (males being hemizygous for the X chromosome) [198]. This correlates with the discovery of twice as many eQTLs in males [152] and greater variability in the expression of X chromosome genes in males [205]. The variation in lifespan associated with autosomes was also found to be greater in males [198]. However, other studies have clearly demonstrated that the range of variability in lifespan in females is wider than that in males [206]. This is in line with data showing that inbreeding greatly reduces lifespan in females, thus bringing females’ lifespan closer to that of inbred males and conversely, outbreeding leads to a greater increase in the lifespan of females, thereby widening the gap between male and female lifespan values [207]. Thus, at present, there is no definitive answer as to which sex of *Drosophila* is more variable in lifespan. However, it is clear that the effect of inbreeding, leading to a decrease in lifespan, is more pronounced in the homogametic sex, i.e., in females. 

It is particularly noteworthy that differences in lifespan between males and females exist in inbred lines—when the lines have the same genetics, that is, they have the same set of alleles (excluding the karyotypic variation in males due to the Y chromosome) [208,209]. This fits well with the idea that sex differences in lifespan in males and females can be controlled by different sets of genes [210]. Modern screening studies have revealed that 1000–2000 genes may be involved in sex-specific variations in lifespan [2]. 

However, when studying individual QTLs associated with lifespan, conflicting results have been obtained. For example, some authors have observed QTL effects associated with sex [164,166,168,173,189,211], including in lines selected for late fecundity and increased lifespan (“O” lines) [171], while other authors have noted the absence of such effects; that is, QTLs affected lifespan in males and females equally [184,186,187]. Furthermore, many of the detected sex-specific QTLs could be a consequence of errors in statistical analysis [212] or a result of keeping males and females separate. The sex-specific effects of these QTLs disappeared when flies were maintained in mixed-sex populations [213]; in other words, when flies had the opportunity to mate. Thus, the results regarding sex-specific QTLs for lifespan are inconclusive. 

When comparing females and males, the expression of 57.5% of genes was found to be sex-dependent [214]. Approximately 4000 genes show more than a two-fold difference in expression between males and females [215]. This correlates well with data from natural populations, in which the majority of detected QTLs were shown to be sex-specific (about 2000 genes) and can exhibit dominance in heterozygotes [152]. Thus, although genes that are differentially expressed in males and females have generally been identified, it is not understood which of them are responsible for differences in the lifespan between males and females. Moreover, the expression of sex-specific genes in hybrids significantly changes depending on the combination of genotypes obtained from crosses between different inbred lines [216].

In addition to differences in gene expression, an additional layer of complexity is imposed by the presence of sex-specific alternative splicing and sex-specific mRNA isoforms for numerous genes, not solely those in the sex determination cascade [217]. The identification of sets of sex-specific genes is complicated by the fact that gene sets differentially expressed in males and females and regulated by a single sex-determining transcription factor (such as Doublesex) may vary depending on the genotype of the line [218]. Thus, the genotype can influence not only the level of expression of sex-specific genes but also the actual set of these genes. Hence, the absence of a clear sexual signature complicates the understanding of which sex-specific genes are responsible for lifespan differences between males and females. It is only clear that additional expression of Doublesex and Fruitless, which regulate sets of genes associated with sex, can change lifespan in both sexes [210].

Validation of the sex-specific influence of individual genes on lifespan is also delayed; however, some clear examples of sexual variability in lifespan exist. One such example is variations in the promoter of the gene encoding for the α-subunit of ATP synthase, leading to an increased level of expression of this gene in males compared to females, resulting in a corresponding sharp decrease in their lifespan [194].

To summarize, it can be noted that although, of course, differences in lifespan between males and females exist, there is still no clear understanding of the mechanisms of the influence of sex on the lifespan of flies. However, the outlines of the reasons for such differences have recently become quite evident. Obviously, there are at least four reasons for differences in lifespan between males and females related to sexual differentiation: (i) the influence of genotype/karyotype—males have one X chromosome, therefore all recessive and epigenetic mutations on the X chromosome manifest in them, (ii) expression of genes of the sex chromosome is generally unbalanced [219,220], (iii) the primary and phenotypic determination of sex, mediated by the *Sxl*→*tra*→*dsx*-*fru* cascade in somatic and *Sxl* in germline cells, leads to differential gene expression in males and females [221,222], and (iv) circulation of hormones primarily related to reproduction affects energy metabolism and the InR insulin/insulin-like growth factor (IGF-1)→target of rapamycin (mTOR) signalling pathway, and through them affects the lifespan [223,224].

### 3.5. Influence of Environmental Factors on the Drosophila Lifespan

In experiments studying lifespan, even under standardized conditions of keeping *Drosophila* in the laboratory, it is impossible to completely exclude the influence of environmental factors such as the social environment, mating, and reproduction on lifespan. Therefore, in this part, we will briefly consider the influence of these environmental factors. 

Several detailed studies have examined the influence of the social environment on the lifespan of flies. In situations where virgin males or females were placed together with competing flies of the same sex (either related or unrelated), the lifespan significantly decreased for virgin males but not for virgin females. However, when virgin males and females were housed separately without competitors, they did not differ in average lifespan [207]. It follows that the effect of the social environment is practically absent in females (Figure 4). However, other studies have revealed that maintaining both virgin and mated females and males, both in groups of the same sex and individually, leads to significant differences in lifespan between males and females [206,208]. Thus, the social environment may affect lifespan, but the direction of this effect seems to depend on the genotype of the strain.

When males and females were kept together in mixed groups, their lifespan was substantially equalized [206,208]. This effect was typical both for flies of inbred lines [206,208] and for flies from natural populations [225]. The equalization of males’ and females’ lifespans in mixed-sex groups results from the activation of at least two natural mechanisms that affect the physiology of flies in relation to reproduction. These are as follows:

(i) Mating increases egg production by females, thus increasing the organism’s expenditure on their production [226,227]. It correlates well with the notion that upregulation of the insulin pathway leads to a decrease in lifespan [3]. The absence of the germline leads to changes in the expression of genes involved in nutrient homeostasis [228] and in classical experiments performed on *D. subobscura*, both virgin females and females without a germline lived significantly longer than fertilised females and the partial reduction of ovaries through heat shock treatment (at 30.5 °C) for 8 days following emergence from pupae (from day 6 to day 8) reduced egg-laying rates in females and increased female lifespan at 20 °C, without affecting males [229]. However, in experiments conducted on *D. melanogaster*, germline ablation unexpectedly increased the lifespan of males but not females [210].
(ii) Mating reduces the lifespan of one sex of *Drosophila* (more often females) [208,230] but lengthens the lifespan of the other (more often males) [226] (Figure 4). The decrease in lifespan in mating females may be due to the non-sperm components of the ejaculate [231,232,233] and is accompanied by changes in gene expression in females depending on the number of matings [234]. The more partners and mating events females engaged in, the greater the reduction in their lifespan [230]. Female diet, including calorie restriction, modifies the presence, magnitude, and direction of the effects of the non-sperm components of the ejaculate on the number of eggs and lifespan, depending on the genotype [235]. Meanwhile, there are examples in the literature where mating dramatically reduces lifespan in both sexes [236], significantly increases lifespan in females [237], or, conversely, reduces lifespan in males depending on the number of females they mate with [238]. The genotype of the crossed lines also plays a significant role. For instance, females from the “late” line show no considerable decrease in lifespan when crossed with males of the “early” line, whereas females from the “early” line exhibit such a decrease [75]. A more in-depth analysis reveals that mating increases lifespan for only six of 25 genotype combinations, and in the remaining cases, mating reduces the lifespan in males and females [237]. Furthermore, studies not conducted in a laboratory but on flies in the wild have revealed that mated females live longer than virgin ones [239]. However, this may, in turn, be due to the fact that the lifespan of flies increases significantly when they are kept in large containers where flight is possible [240,241]. This correlates with the classical data showing that flies with wings removed upon emerging from pupae have a shorter lifespan than normal ones [32]. In experiments conducted by Markow [239], newly emerged females were compared with fertilised females that had been captured nearby and had the opportunity to fly beforehand. However, wing removal could also affect other functions or simply be traumatic.

Thus, it is now clear that reproduction and social environment can influence the lifespan of *Drosophila*. Keeping males and females in a mixed population leads to a significant equalisation of their lifespans, apparently not due to a negative effect of non-sperm components or increased egg production on the females, but due to a general change in gene expression caused by mating in males and females [242], and a corresponding change in physiology in both sexes. Although the conditions for keeping males and females together are more “physiological” for flies and more similar to conditions in nature, keeping males and females together in longevity studies requires the sex of the dead flies to be determined, so studies where virgin males and females are kept separately dominate the literature. Regarding the influence of the social environment on lifespan, the mechanism behind this effect is not entirely clear. A precise understanding of the conditions under which, and the circumstances in which, this occurs requires further clarification. 

### 3.6. The Role of Stochastic Variability in Lifespan Determination

Interestingly, in some laboratory experiments, researchers were unable to reproduce the effects of sex, mating, and reproduction, but they reliably observed effects in response to changes in laboratory housing conditions, fly feeding, and genetic background of the line [243]. The authors concluded that the stochastic component significantly influences the lifespan of flies. This impact on individual development, sometimes referred to as microenvironmental plasticity (the term may not be entirely fitting, given that laboratory research is conducted under highly uniform environmental conditions, and “plasticity” typically refers to variability in phenotype), arises from stochastic variations in gene expression [244]. Accordingly, the determination of individual transcriptomes of flies kept under strictly controlled conditions, with the same mating status and sex, revealed that despite inbred genotypes and meticulously controlled environmental conditions, thousands of genes were differentially expressed between individuals (up to 23% of genes), and the level of expression variability between individual flies depended on the line genotype [244]. Unfortunately, this study did not examine the relationship between stochastic gene expression and lifespan in specific genotypes. However, studies on self-fertilizing worms like *C. elegans*, for which generating a series of isogenic clones is straightforward, have shown that such clones also exhibit random, unrelated to genetic or environmental factors, variability in the expression levels of certain genes—such as those involved in the response to heat shock [245]. Indeed, this level of variability affects both the worms’ resistance to overheating and their lifespan: clones with higher levels of heat shock protein expression tend to live longer. It was shown that differences in the degree of expression of heat shock genes existed in genetically identical worms even before they were subjected to separation according to the heat shock gene expression level, and subsequent analysis of heat resistance and lifespan. Individuals that responded more actively to heat shock gained an advantage under overheated conditions. However, mutations leading to increased expression of heat shock genes are not fixed in the worm’s gene pool, as individuals with a strong stress response have reduced fertility [245]. In the population, it appears advantageous to have two types of worms: rapidly reproducing individuals that are stress-sensitive, and less fertile but stress-resistant ones. Thus, worms minimize the risk of extinction by allowing a wide variability in the expression of heat shock genes even among genetically identical individuals. Random variation in gene expression may itself be an adaptation favoured by selection in worm populations, as natural populations of *C. elegans* often exhibit low genetic diversity due to the small number of founders and the resultant mechanism of self-fertilisation [246]. On the other hand, it should be noted that genetically diverse populations typically exhibit a different pattern. In such populations, high variability is a characteristic feature of traits and is not subject to selection pressure, at least not to a strict one [201]. The high variability indicates that the trait does not affect fitness (the survival and reproductive success of individuals), so a wide range of trait variants freely emerges in the gene pool.

Therefore, undoubtedly, weakened selection pressure on lifespan can be considered a cause of high variability in the lifespan trait in natural populations of *Drosophila*. However, in genetically homogeneous populations, the source of genetic variability for the lifespan trait is limited.

Thus, genetically identical worms, like flies, exhibit random variability in gene expression levels. Stochastic fluctuations at the molecular level, including variations in gene expression, may play a significant role in lifespan variability. The extent of this contribution remains to be determined.

Specific molecular mechanisms may underlie a gene’s “propensity” to vary in expression. For instance, an increase in regulatory complexity can translate into a more stable and unchanging expression. For example, using expression data from 75 isogenic *Drosophila* lines, genes with low variability were determined to fall into two classes reflecting different mechanisms for maintaining stable expression, while genes with high variability appeared to lack both stabilising mechanisms [247]. The first stabilizing mechanism involves the presence of broad promoters with multiple transcription initiation sites and high occupancy of transcription factors. The second mechanism entails narrow promoters with paused RNA polymerase II and high regulatory complexity beyond their promoter region. By contrast, genes with high expression variability tend to have narrow promoters and few regulatory features, suggesting that the absence of transcriptionally “stabilising” mechanisms may contribute to a wide variation in their expression. Thus, the structural organization features of genes likely underlie the stochastic component of variability observed in genetically homogeneous *Drosophila* populations, including lifespan.

### 3.7. Lifespan Trait Controlled Simultaneously by Different Types of Variability 

**Figure 5:**
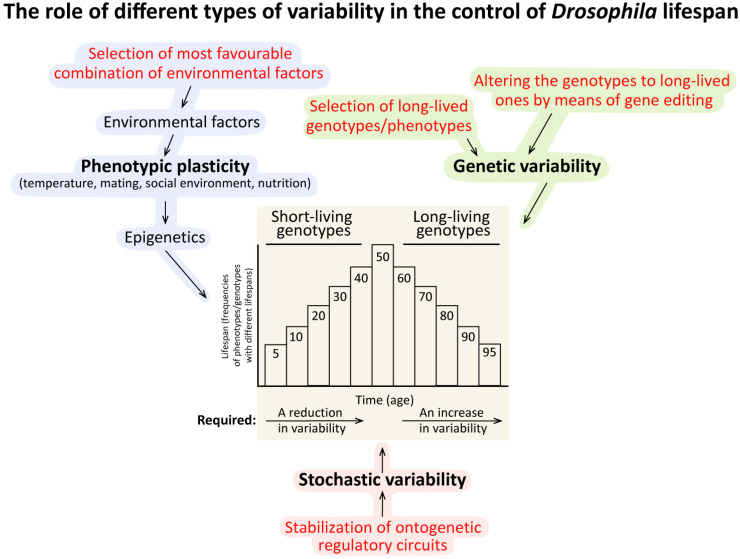
The role of different types of variability in the control of *Drosophila* lifespan. The frequencies of long- and short-living phenotypes in the population vary depending on three types of variability (highlighted in bold text). Strategies to influence these three types of variability are indicated in red. To shift the values of the lifespan trait distribution to the right, it is necessary to reduce variability on the left and increase it on the right. An alternative approach (not shown) is to decrease variation on both sides of the distribution (i.e., reduce the reaction norm), and then shift the values of the lifespan trait distribution to the right using one of the strategies indicated in red. The X-axis represents time (age) in days, while the Y-axis represents a distribution showing the frequencies of phenotypes/genotypes with different lifespans.

Variation in the lifespan trait, like all other traits, is influenced by three types of variability. The most obvious and, at first glance, simplest form of variation is lifespan variability caused by the influence of environmental factors. It is evident that selecting an optimal combination of such factors will, up to a certain limit, increase the average lifespan. This means that it will probably not be possible to cross the limits to maximum lifespan of the species using these methods. On the other hand, the phenotypic plasticity of an organism is realised through changes in epigenetic factors (Figure 5). Active research into epigenetics has only recently begun, so the potential of this type of variability may be underestimated. For example, the epigenetic (information) theory of ageing has recently emerged, according to which the cause of ageing is the blurring over time of the correct epigenetic features of the regulation of genome expression [248]. This effect is often caused by the disruption of epigenetic information even during error-free DNA replication [249]. There are great hopes for so-called epigenetic rejuvenation [250,251,252,253,254]. However, certain problems accompany this approach, such as organ dysfunction and tumour development [251,255].

Genetic (heritable) variability is the most apparent yet also the most enigmatic form of variability (Figure 5). This is due to the difficulty in identifying genetic variations that can increase lifespan, as well as the possibility of epistatic interactions between gene variants, where variations influence each other’s expression. Solving the issue of genetic variability in controlling lifespan may involve, to some extent, selecting genetically compatible pairs of individuals during genetic counselling (when using reproductive technologies, selecting variants conducive to longevity may be possible), as well as utilizing gene editing technologies to introduce variations associated with increased lifespan into the human genome (for example, those characteristic of supercentenarians). Currently, the general prohibition on editing human zygotes and embryos precludes such manipulations at early stages of development, but some successes in somatic cell editing in vivo and ex vivo offer hope that some progress using gene editing will still be achieved.

The most intricate form of variability is stochastic. Advancements in technology have enabled the study of the molecular basis of lifespan at the individual level. It has been demonstrated that a considerable portion of lifespan variation stems from differences in gene expression among genetically identical individuals, influenced by the structure of genes themselves and the organization of their regulatory networks. Mitigating intrinsic variation in gene expression and bolstering regulatory networks poses significant challenges for the future (Figure 5). Addressing the latter issue requires extensive advancements in gene editing technologies. Research aimed at resolving the issue of stochastic variability may not be feasible until at least the middle of this century, pending the development of corresponding technologies.

## 4. Concluding Remarks, Future Problems, and Perspectives

To date, the history of using *Drosophila* in research spans more than 110 years. At the beginning of the last century, the foundations for the genetics of longevity were laid, and it was demonstrated that lifespan behaves like a typical Mendelian trait. In particular, it was shown that mutations can both shorten and lengthen how long the flies live. These early findings made it evident that genetic variation plays a significant role in determining longevity. Experiments continued to explore the possibilities of lifespan selection. Two main selection methods were used: selection for late fecundity and direct selection for long-lived flies. These studies were successful, demonstrating the feasibility of controlling longevity in a genetic experiment. Several long-lived, genetically heterogeneous *Drosophila* lines were obtained. It has been shown that the response to selection depends not only on the method and duration of selection, but also on factors such as larval population density (contribution of environmental factors) and the initial genetic diversity of the original fly population. In parallel, attempts were made to search for specific genetic DNA variations associated with increased longevity (longevity QTLs). Unfortunately, due to insufficient statistical power and the limited availability of sequenced inbred fly lines, the accuracy of identifying genes responsible for lifespan QTLs is very limited.

Genetic diversity arises from the presence of multiple variants of a gene, known as alleles, within the genetic pool. These genetic polymorphisms can alter gene expression levels, and selection for longevity induces changes in transcriptional responses in both young and aged flies. It would be extremely intriguing to uncover the specific DNA changes and polymorphisms responsible for the characteristic transcriptional responses to selection for increased lifespan with an extended reproductive period in flies. Considering that complex phenotypes, both at the molecular and organismal levels, may be underpinned by a limited, perhaps very small number of genetic changes [203,246], the number of “guilty” DNA changes responsible for increasing lifespan and reproductive period could be exceedingly small, perhaps just a few. However, the genetic heterogeneity of long-lived lines and the multitude of mutations identified within them [100], along with the extensive number of genes exhibiting altered expression in response to selection for late fecundity [100,101], may hinder obtaining this answer using existing long-lived fly lines. All of this indicates the necessity in the coming years to develop new schemes for selection for increased lifespan, preferably rapid, and to breed new long-lived lines, preferably inbred to reduce the number of neutral polymorphisms [256].

Studies examining genetic variation in longevity, which began in the early 20th century, have been accompanied by investigations into the phenotypic plasticity of the longevity trait influenced by environmental factors such as temperature conditions, feeding, social interactions, mating, and reproduction. These studies persisted throughout the entire 20th century. Temperature emerged as the strongest factor influencing phenotypic plasticity in lifespan. High temperatures were found to have a negative effect on adult fly lifespan and female fecundity. With regard to nutrition, studies showed that overeating reduced lifespan, leading to extensive research into the effects of calorie restriction on lifespan in *Drosophila* and other model organisms. The general consensus from these many studies was that the interaction between genetic background and temperature conditions had a more significant effect on lifespan than factors related to reproduction/mating or the social environment. 

Stochastic variability is often underestimated and may play a crucial role in lifespan control. Absolutely, more research is essential for gaining a deeper understanding and exploring this type of variability.
